# Simulation of Genomes: A Review

**DOI:** 10.2174/138920208784340759

**Published:** 2008-05

**Authors:** Antonio Carvajal-Rodríguez

**Affiliations:** Departamento de Bioquímica, Genética e Inmunología, Universidad de Vigo, 36310 Vigo, Spain

## Abstract

There is an increasing role of population genetics in human genetic research linking empirical observations with hypotheses about sequence variation due to historical and evolutionary causes. In addition, the data sets are increasing in size, with genome-wide data becoming a common place in many empirical studies. As far as more information is available, it becomes clear that simplest hypotheses are not consistent with data. Simulations will provide the key tool to contrast complex hypotheses on real data by generating simulated data under the hypothetical historical and evolutionary conditions that we want to contrast. Undoubtedly, developing tools for simulating large sequences that at the same time allow simulate natural selection, recombination and complex demography patterns will be of great interest in order to better understanding the trace left on the DNA by different interacting evolutionary forces. Simulation tools will be also essential to evaluate the sampling properties of any statistics used on genome-wide association studies and to compare performance of methods applied at genome-wide scales. Several recent simulation tools have been developed. Here, we review some of the currently existing simulators which allow for efficient simulation of large sequences on complex evolutionary scenarios. In addition, we will point out future directions in this field which are already a key part of the current research in evolutionary biology and it seems that it will be a primary tool in the future research of genome and post-genomic biology.

## INTRODUCTION

There is an increasing role of population genetics in human genetic research linking empirical observations with hypotheses on sequence variation due to historical and evolutionary causes. In addition, the data sets are increasing in size, with genome-wide data becoming a common place in many empirical studies [1]. As far as more information is available, it becomes clear that simplest hypotheses (neutrality, constant population size, uniform recombination) are not consistent with data. Therefore, to understand the trace left in the DNA by historical and evolutionary factors, more complex predictive hypotheses are needed. Simulations will provide the key tool to contrast complex hypotheses on real data by generating simulated data under the hypothetical historical and evolutionary conditions that we want to contrast. Thus, we can distinguish among models by simulating their evolutionary consequences concerning a given hypothesis [1, 2].

Currently, one of the most exciting examples of the importance of a population perspective in human genetics is the study of patterns of linkage disequilibrium (LD) in humans [3]. The knowledge of such patterns will facilitate the assembly of genome haplotype maps [4-6] improving enormously the efficiency of disease gene mapping. It seems that these blocks are mainly defined by recombination hot spots [7, 8]. However, haplotype blocks can also be generated by genetic drift in regions of uniform recombination provided this is low enough [9]. Therefore, we have now growing empirical knowledge about haplotype block and tagSNPS diversity but less is known about the effect of population demographic factors. We have no clear ideas on how the combined effect of genetic drift, mutation, recombination and migration, affect LD and tagSNP patterns though is known they do [10]. Computer simulations will provide a powerful tool to test different hypotheses, allowing the disentanglement of complex evolutionary patterns that will be difficult to understand in any other way. For example, the history of past human migration provides important clues to understand present patterns of human DNA variation. Computer simulations have already provided important information to test hypotheses concerning population histories [11, 12].

The growing importance of simulations to fulfill the needs for more complex models to explain current DNA patterns is reflected by the increase of efficient computer simulation programs that aim to deal both with high amount of data and with complex models of evolution. Certainly, the development of tools to simulate large sequences under natural selection, recombination and complex demographic patterns is already of great interest in order to better understand the signal left on the DNA by different interacting evolutionary forces. Simulation are already, and will continue to be, an essential tool to evaluate the sampling properties of any statistics used on genome-wide association studies and to compare performance of methods applied at genome-wide scales. Thus, there are two main different approaches of computer simulation in population genetics, namely, backward or forward strategies can be followed. Both kinds of strategies are complementary. Several new recent simulation tools, both backward and forward, have being developed. We aim to review some of the recently developed simulators which allow for efficient simulation of large sequences on complex evolutionary scenarios. In addition, we will point out future directions in this field which are already a key part of the current research in evolutionary biology and it seems that it will be a crucial issue in the future research of genome and post-genomic biology.

Noteworthy, in this review we do not intend to mention every program that can simulate the evolution of genetic information because that list will be enormous and is increasing each day. We will mention just some programs that, firstly, provide enough information to allow friendly use for a non-programmer person and, secondly, can simulate in an efficient way medium or long fragments of DNA e.g. at least 1 megabase of DNA in the case of coalescent programs or 10^3^ unlinked genes in the case of forward simulators. By efficiency we mean simply the speed of a computational process in a one-processor system.

## COALESCENT SIMULATORS

Coalescence is a sample-based theory relevant to the study of population samples and DNA sequence data [13-15]. A random genealogy of a sample is generated and then mutations are randomly placed on the genealogy. Thus, coalescent-based simulations, are computationally very efficient because they are backward based on the history of lineages with survived offspring in the current population ignoring, however, all those whose offspring did not arrived to the present [16]. Due to its efficiency, it has been used to derive several algorithms to estimate parameter values that maximize the probability of the given data [17].

In Table **1** we can see different coalescent simulators that somewhat allow efficient simulation of more or less large DNA fragments evolving under complex evolutionary models. The most classical one, ms [18], permits flexible and efficient simulation of different standard neutral evolutionary models with recombination, variable population size, migration, etc. Thus, ms program can efficiently generate samples (only with 2-allele segregating sites) and trees under different neutral scenarios. Different programs focus different effort in modelling distinct and specific aspects of evolution. For example, SPLATCH [19] allows modelling spatial and temporal environmental heterogeneity, while SelSim [20] allows to study the combining effect of selection and recombination and the Fearnhead set of programs [21] allows the study of the impact of strong selection onto patterns of variability under different scenarios. Noteworthy, coasim [22], cosi [1], msHOT [23], mlcoalsim [24] and GENOME [25] programs allow for a flexible and complete set of situations including recombination hotspots. Finally, the efficiency of the programs is very important because will allow to simulate larger sequences in acceptable times. Thus, efficiency, i.e. the speed of the process should be a consequence of better algorithms that allow for both a good use of computer memory space and faster execution times. In this aspect some programs were noticeable. For instance, various programs need about 10-15 minutes to simulate 10,000 samples of size 100 chromosomes with 250 SNPs each (or a DNA region with 250 partially linked loci) under a population size of 1,000 and a population recombination rate of 10 for the whole chromosome segment. These settings imply about a genome segment of 250 Kb assuming 1cM per Mb. However, mlcoalsim [24] and Coasim [22] take seconds. Unfortunately, mlcoalsim does not produce “real” sequences because just manage two allele variants per site. The program is anyway very useful for testing hypotheses e.g. demography and selection impacts on linkage disequilibrium at the genome level [26]. Another program that is very efficient is Seq-Gen [27] which produces samples of length 10Mb in seconds. However, in the case of Seq-Gen the user needs a phylogenetic tree to evolve the sequences along the phylogeny. It does not assume recombination but different data partitions can be made with different trees. It can be piped with the output of other programs as ms. A similar program to Seq-Gen is Evolver which belongs to package PALM [28] however to change some of the options in Evolver the user needs to change the source code and recompile.

Indeed, the need is increasing of simulating larger DNA regions under complex evolutionary situations. Fortunately, some new algorithms are also emerging. Noticeable is GENOME [25] which uses a modified coalescence algorithm to allow for the impressive numbers of 150 Mb in 1 hour managing scaled mutation, recombination and migration rates of the order of 6 × 10^4^. Other important new method is fastcoal [29] which uses a new algorithm for fast coalescent simulation of large DNA segments, being able to simulate genome-wide data several orders of magnitude faster than classical coalescent ones. However, fastcoal makes simplifying assumptions about the genealogy that GENOME does not.

## FORWARD SIMULATORS

Forward simulations are less efficient than coalescent based ones because the whole history of the sample is followed from past to present. On the other hand, forward simulation has some advantages over the coalescent framework. The first of all is the same that causes coalescent simulation efficiency, namely, the coalescence does not keep track of the complete ancestral information. In consequence, if the interest is focused on the evolutionary process itself, rather than on its outcome, forward simulations should be preferred [33]. Second, coalescent simulations are complicated by simple genetic forces such as selection, and although different evolutionary scenarios have been built-in (see Table **1** above) it is still difficult to implement models incorporating complex evolutionary situations with different kinds of selection, variable population size, recombination, complex mating schemes, and so on. In fact, we can only simulate very limited forms of selection and recombination under the coalescent. In addition, when simulating recombination under a coalescent codon model we usually do not account for intracodon recombination. Similarly, coalescent methods cannot yet simulate realistic samples of complex human diseases [34]. Indeed, when simulating non-neutral scenarios and/or complex models under the coalescence, much of the computational efficiency is lost. Moreover, the coalescent model is an approximation based on specific limiting values and relationships between some important parameters [35]. Hence, there is an increasing interest on forward population genetic simulation and new efficient tools have been developed recently. In Table **2** some of these forward simulators are listed. The oldest ones are FPG [36] and EASYPOP [37]. FPG can simulate a broad range of conditions including natural selection, recombination migration and so on. However is somewhat limited by the genome size it can manage. It allows for a total genome length of up to 1000 segments each limited to 32 polymorphic sites. With these lengths, could be possible to model a genome of up to 3.2 Mb. However with high population sizes and genome lengths the program is very slow. EASYPOP has a more efficient use of memory (can manage thousands of SNPS) but simulates only neutral loci. More powerful are some new forward simulators that recently emerged. For example, SIMUPOP [38] can manage large multi-generation populations with mutation, migration and selection hence allowing user-defined disease allele frequencies. However, running such complex models require that the user write its own macros in the python language. Other new flexible forward simulators are FREEGENE [39] and GenomePop [40] which use techniques as scaling to simulate large populations and genomic regions through many generations. FREEGENE permits both directional and balancing selection but manage only two allele models and symmetric Island migration model. GenomePop permits only directional selection, but real DNA sequences and arbitrary migration models.

These programs can manage a high number of independent or linked SNPs. For example, FREEGENE is able of simulate genome regions of several Mb during 10*N* generations in a personal computer in acceptable time. GenomePop is also able to evolve a genome of 100 chromosomes with 1,000 SNPs each. Considering uniform recombination of 0.1 per genome (population recombination rate of 40 per chromosome) and assuming 1cM per 1Mb this implies 0.1 Mb per chromosome i.e. a 10 Mb genome. 

## CONCLUSIONS

Simulation software is already a key part of the current research in evolutionary biology and it will be a primary tool in the future research of genome and post-genomic evolutionary biology (Table **3**). The feasible understanding of evolutionary processes will provide humans with the tools to meliorate human health and fitness. The future should find us in the effort of combining the insight provided by complex stochastic models with the thoughtful use of simulation methods for both, inference and modelling of complex evolutionary scenarios. Therefore, more sophisticated algorithms will be developed to represent and simulate efficiently the genetic data. Hence, the efficiency of new algorithms jointly with the use of multiple-computer clusters will hopefully allow the study of the virtual evolution of genomes under very different conditions.

## Figures and Tables

**Table 1.** Different Coalescent Simulators for Genomes Evolving Under Complex Evolutionary Models. The Programs are Sorted by the Reference Date

Name	Sel	Rec	VRec	VarN	M	MM	CEM	Tree	Ref
Seq-Gen	No	No	No	No	No	Yes	Yes	Yes	R97
TREEVOLVE	No	Yes	No	Yes	Yes	No	No	No	G99
SIMCOAL2	No	No	No	Yes	Yes	Yes	No	Yes	E00
ms	No	Yes	No	Yes	Yes	No	No	Yes	H02
SPLATCHE	No	No	No	Yes	Yes	Yes	No	Yes	C04
SelSim	Yes	Yes	No	No	No	Yes	No	No	S04
Serial SIMCOAL	No	No	No	Yes	Yes	Yes	No	Yes	A05
Coasim	No	Yes	Yes	Yes	Yes	Yes	No	No	M05
Cosi	No	Yes	Yes	Yes	Yes	Yes	No	No	S05
Hap and dip	Yes	No	No	Yes	Yes	Yes	No	No	F06
msHot	No	Yes	Yes	Yes	Yes	No	No	Yes	He07
GENOME	No	Yes	Yes	Yes	Yes	No	No	Yes	L07
mlcoalsim	Yes	Yes	Yes	Yes	Yes	No	No	No	R07
Evolver	No	No	No	No	No	Yes	Yes	Yes	Y07

Sel: Selection. Rec: recombination. VRec: Variable recombination rates. Var N: Variable population size. M: Migration. MM: Different Mutation Models. CEM: Codon or amino-acid evolution models. Tree: Produces a genealogy. Ref: Reference. R97: [27]. G99: [30]. E00: [31]. H02: [18]. C04: [19]. S04: [20]. A05: [32]. M05: [22]. S05: [1]. F06: [21]. He07: [23]. L07: [25]. R07: [24]. Y07: [28].

**Table 2.** Different Forward Simulators for Genomes Evolving Under Complex Evolutionary Models. The Programs are Sorted by the Reference Date

Name	Sel	Rec	VRec	VarN	M	MM	CEM	Seq	Tree	Ref
FPG	Yes	Yes	No	No	Yes	No	No	Yes	No	JH
EasyPop	No	Yes	No	No	Yes	Yes	No	No	Yes	B01
SimuPop	Yes	Yes	Yes	Yes	Yes	Yes	No	No	Yes	P05
FREEGENE	Yes	Yes	Yes	Yes	Yes	No	No	No	No	H07
GenomePop	Yes	Yes	Yes	Yes	Yes	Yes	Yes	Yes	No	C08

Sel: Selection. Rec: recombination. VRec: Variable recombination rates. Var N: Variable population size. M: Migration. MM: Different Mutation Models. CEM: Codon Evolution Model. Seq: The user gets a DNA sequence sample. Tree: The user gets the genealogy of the sample. Ref: Reference. JH: [36]. B01:[37]. P05: [38]. H07: [39]. C08: [40].

**Table 3.** Web Links to the Programs Cited in this Review Sorted in Alphabetical Order

Name	Web Link
Coasim	http://www.daimi.au.dk/~mailund/CoaSim/
Cosi	http://www.broad.mit.edu/~sfs/cosi/
EasyPop	http://www.unil.ch/izea/softwares/easypop.html
Evolver	http://abacus.gene.ucl.ac.uk/software/paml.html
FPG	http://lifesci.rutgers.edu/~heylab/HeylabSoftware.htm#FPG
FREEGENE	http://www.ebi.ac.uk/projects/BARGEN/download/FREGEN/
GENOME	http://www.sph.umich.edu/csg/liang/genome/
GenomePop	http://webs.uvigo.es/acraaj/GenomePop.htm
hap	http://www.maths.lancs.ac.uk/~fearnhea/software/PS.html
mlcoalsim	http://www.ub.edu/softevol/mlcoalsim/
ms	http://home.uchicago.edu/~rhudson1/source/mksamples.html
msHot	http://home.uchicago.edu/~rhudson1/source/mksamples.html
PALM	http://abacus.gene.ucl.ac.uk/software/paml.html
SelSim	http://www.stats.ox.ac.uk/~spencer/SelSim/Controlfile.html
Seq-Gen	http://tree.bio.ed.ac.uk/software/seqgen/
SerialSIMCOAL	http://iod.ucsd.edu/simplex/ssc/
SIMCOAL2	http://cmpg.unibe.ch/software/simcoal2/
SimuPop	http://bp6.stat.rice.edu:8080/simuPOP/
SPLATCHE	http://cmpg.unibe.ch/software/splatche/
TREEVOLVE	http://evolve.zoo.ox.ac.uk/software.html?name=Treevolve
